# Transcriptomic analysis of *Macrobrachium rosenbergii* (giant fresh water prawn) post-larvae in response to *M. rosenbergii* nodavirus (*Mr*NV) infection: de novo assembly and functional annotation

**DOI:** 10.1186/s12864-019-6102-6

**Published:** 2019-10-22

**Authors:** Phongthana Pasookhush, Charles Hindmarch, Paisarn Sithigorngul, Siwaporn Longyant, William G. Bendena, Parin Chaivisuthangkura

**Affiliations:** 1 Department of Biology, Srinakharinwirot University, Sukhumvit 23, Bangkok, 10110 Thailand; 2 QCPU, Queen’s Cardiopulmonary Unit, Translational Institute of Medicine (TIME), Department of Medicine, Queen’s University, Kingston, ON K7L 3N6 Canada; 3 Center of Excellence for Animal, Plant and Parasite Biotechnology, Srinakharinwirot University, Sukhumvit 23, Bangkok, 10110 Thailand; 4 Department of Biology, Queen’s University, Kingston, ON K7L 3N6 Canada

**Keywords:** *Macrobrachium rosenbergii*, *M. rosenbergii* nodavirus, Crustacean immunity, RNAseq, de novo transcriptome assembly, Differential expression analysis

## Abstract

**Background:**

*Macrobrachium rosenbergii*, is one of a major freshwater prawn species cultured in Southeast Asia. White tail disease (WTD), caused by *Macrobrachium rosenbergii* nodavirus (*Mr*NV), is a serious problem in farm cultivation and is responsible for up to 100% mortality in the post larvae stage. Molecular data on how *M. rosenbergii* post-larvae launches an immune response to an infection with *Mr*NV is not currently available. We therefore compared the whole transcriptomic sequence of *M. rosenbergii* post-larvae before and after *Mr*NV infection.

**Results:**

Transcriptome for *M. rosenbergii* post-larvae demonstrated high completeness (BUSCO Complete: 83.4%, fragmentation: 13%, missing:3.3%, duplication:16.2%; highest ExN50 value: 94%). The assembled transcriptome consists of 96,362 unigenes with N_50_ of 1308 bp. The assembled transcriptome was successfully annotated against the NCBI non-redundant arthropod database (33.75%), UniProt database (26.73%), Gene Ontology (GO) (18.98%), Evolutionary Genealogy of Genes: Non-supervised Orthologous Groups (EggNOG) (20.88%), and Kyoto Encyclopedia of Genes and Genome pathway (KEGG) (20.46%). GO annotations included immune system process, signaling, response to stimulus, and antioxidant activity. Differential abundance analysis using EdgeR showed 2413 significantly up-regulated genes and 3125 significantly down-regulated genes during the infection of *Mr*NV.

**Conclusions:**

This study reported a highly complete transcriptome from the post-larvae stage of giant river prawn, *M. rosenbergii.* Differential abundant transcripts during *Mr*NV infection were identified and validated by qPCR, many of these differentially abundant transcripts as key players in antiviral immunity. These include known members of the innate immune response with the largest expression change occurring in the *M. rosenbergii* post-larvae after *Mr*NV infection such as antiviral protein, C-type lectin, prophenol oxidase, caspase, ADP ribosylation factors, and dicer.

## Background

*Macrobrachium rosenbergii*, known as the giant freshwater prawn, is an economically important crustacean species in Southeast Asia. Production of *M. rosenbergii* increased dramatically over the last 20 years and has exceeded 200,000 t per year since 2002 [1]. A major problem in the cultivation of *M. rosenbergii* relates to loss through infectious diseases caused by viruses, bacteria, and fungi [2]. One of the most serious viral threats to *M. rosenbergii* culture is *Macrobrachium rosenbergii* nodavirus (*Mr*NV) that causes white tail disease (WTD). This disease was first discovered in Guadeloupe Island [3] and subsequently in Taiwan [4], China [5], India [6], Thailand [7], and Australia [8]. *Mr*NV is a non-enveloped, icosahedron with 26–27 nm diameter composed of a nucleocapsid bearing two positive single-stranded RNA genomes (RNA-1 and RNA-2). RNA-1 is 3202 bp - ssRNA encoding protein A or RNA-dependent RNA polymerase (RdRp) and protein B2 [9]. Protein B2 is capable of inhibiting the RNAi pathway of the host cell [10]. RNA-2 is 1175 bp - ssRNA encoding capsid protein [11]. The prominent sign of infected post larvae (PL) is the appearance of whitish muscle commonly in abdominal region. Mortalities may reach 100% within 7–15 days after the infection or 3–5 days after the appearance of the first anatomical signs [6]. However, experimental transmission of *Mr*NV revealed that the virus failed to cause mortality in adult prawns [6]. As there is no cure for WTD infected prawns, preventive procedures have been implemented such as screening of brood stock and PL, and good farm management [7, 12, 13]. Understanding the prawn’s immune response specifically to WTD may also provide bio-rationale targets to help contain and restrict disease outbreak.

As an arthropod crustacean, the innate immune system of *M. rosenbergii* is composed of both humoral and cellular responses. Humoral immune responses include up-regulation of the prophenol oxidase system (ProPO), clotting proteins, melanization and antimicrobial peptides [14]. Cellular immune responses involve hemocyte activities such as phagocytosis, apoptosis, nodule formation, and encapsulation which function to neutralize pathogens [15]. Indirect recognition of pathogens or pathogen-associated molecular patterns (PAMPs) by pattern recognition receptors (PRRs) such as Toll receptors leads to the activation of these humoral and cellular immune responses [16].

In recent years, high-throughput technology such as next generation sequencing (NGS) has emerged and is widely used in both genomic and transcriptomic research. NGS technology can also be used to study differential gene-expression on various tissues or certain conditions such as stress and pathogen infection [17] and in both model, and non-model organisms [18]. There are transcriptomic studies for penaeid shrimps such as *Peneaus monodon* [19–21], *Litopeneaus vannamei* [22–24], *Fenneropeneaus chinensis* [25, 26], *Fenneropenaeus merguiensis* [27, 28], and *Marsupeneaus japonicus* [29] to investigate tissue-specific expression, the stress response, and viral infection. Moreover, many studies have been performed on whole transcriptome sequencing of the hepatopancreas of *M. rosenbergii* in response to *Vibrio parahaemolyticus* infection [30], hepatopancreas and lymphoid organ in response to white spot syndrome virus (WSSV) [31, 32], and intestinal tissue in response to WSSV or the viral PAMP mimic (poly I:C) [33]. Most recently, transcriptomic analysis of hematopoietic tissue of *M. rosenbergii* adult prawn in response to *Mr*NV infection has been studied [34]. Many of differentially abundant transcripts were belonged to various immune mechanisms such as pattern-recognition receptors, antioxidants, and antimicrobial peptides [34].

*Mr*NV has direct impact on *M. rosenbergii* post-larvae culture. However, there is no transcriptomic data on *M. rosenbergii* post-larvae in response to the infection with *Mr*NV. To address this deficiency, the whole transcriptome from six biological replicates of each treatment from healthy and *Mr*NV-infected PL were sequenced. In this study, a highly complete transcriptome for *M. rosenbergii* post-larvae was assembled and annotated. This transcriptome can be used as reference transcriptome for the further gene expression analysis of *M. rosenbergii.* Differential abundant transcripts were examined and immune-related genes in response to *Mr*NV infection were reported. In addition, differential abundance and RNAseq results were validated by quantitative PCR in separate biological samples.

## Results

### Sequence read data and raw data pre-processing

RNA sequencing for two groups with six biological replicates (*n* = 12) produced a total of 522,296,142 raw reads with an average of 43.5 ± 5.1 (mean ± SD) million read pairs per sample (Table 1). The raw reads were subjected to quality trimming which included adaptor removal. After quality trimming, a total of 501,900,423 (96.09%) 65 bp trimmed reads had high average Phred score (96.13% with Phred ≥30).

**Table 1:** The number of read pairs prior and after trimming

Sample	Raw paired-end reads	Trimmed paired-end reads
Control 1	46,497,004	44,697,960 (96.13%)
Control 2	43,309,253	41,651,586 (96.17%)
Control 3	40,571,354	38,896,140 (95.87%)
Control 4	42,111,175	40,566,032 (96.33%)
Control 5	47,098,377	45,404,272 (95.63%)
Control 6	38,519,779	36,568,513 (94.93%)
Infected 1	44,325,031	42,803,756 (96.57%)
Infected 2	46,902,950	45,238,551 (96.45%)
Infected 3	49,289,659	47,443,959 (96.26%)
Infected 4	39,687,853	38,299,205 (96.50%)
Infected 5	33,036,002	31,758,938 (96.13%)
Infected 6	50,947,705	48,935,511 (96.05%)
Total reads	522,296,142	501,900,423 (96.09%)

### De novo transcriptome assembly and quality assessment

The trimmed reads were subjected to de novo transcriptome assembly using Trinity. The trimmed reads were further reduced into 51,971,920 reads (10.36%) during in silico normalization prior to de novo assembly. The Trinity assembler produced 109,616 which were then clustered into 96,362 unigenes with N_50_ of 1308 bp, and mean length of 776.73 bp by CD-HIT software. The transcriptome had high overall fragment mapping rate of 96.85% with 90.08% that aligned concordantly ≥1 times. Based on BUSCO, the assembled transcriptome was highly complete with 889 (83.4%) ortholog gene from Arthropoda database with low fragmented and missing BUSCOs (C:83.4%[S:67.2%,D:16.2%],F:13.3%, M:3.3%,n:1066). In addition, the E_x_N_50_ statistics showed that the maximum N_50_ value was on E_94%_ with 2031 bp N_50_ length. The summary of the transcriptome assembly and quality assessment are shown in Table 2.

**Table 2:** Summary of the transcriptome assembly and quality assessment

Prior to de novo assembly	
Length of raw reads (bp)	75
Total number of raw reads	522,296,142
Length of trimmed reads (bp)	65
Total number of trimmed reads	501,900,423 (96.09%)
Total number of normalized reads	51,971,920 (10.36%)
After de novo assembly	
Total number of contigs	109,616
Total number of unigenes	96,362
Mean length of unigenes (bp)	776.73
N_50_ length of unigenes (bp)	1308
Highest E_x_N_50_ value (% Ex)	94
Fragment mapping rate (%)	96.85%
BUSCO completeness (%)	83.4%

### Functional annotation

**Fig. 1:**
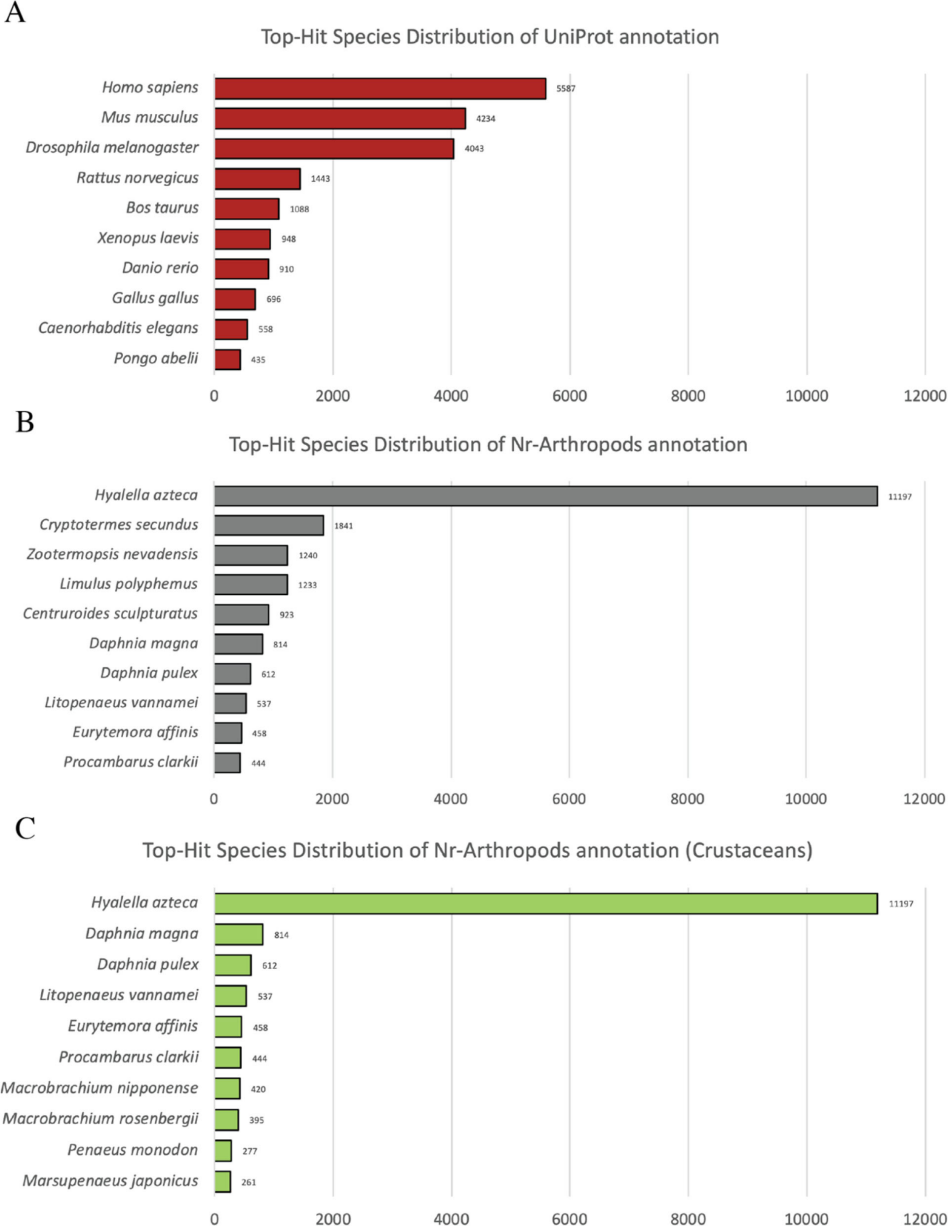
Top 10 species distribution of Blastx results from different databases. **a** UniProt database **b** Non-redundant arthropod database **c** Non-redundant arthropod database with only crustacean species

The assembled transcripts were subjected to the homology search by Blastx software using UniProt and non-redundant arthropod database. Blastx search against UniProt database yielded 25,761 (26.73%) significant hits (*E*-value < 1-e5). The majority of top-hit species distribution were *Homo sapiens* with 5587 (21.7%) hits followed by *Mus musculus* and *Drosophila melanogaster* with 4234 (16.4%) and 4043 (15.7%) hits, respectively (Fig. 1a).

In case of non-redundant arthropod database, Blastx search yielded 32,523 (33.75%) significant hits (*E*-value < 1-e5). Top-hit species distribution was mainly dominated by *Hyalella azteca* with 11,197 (34.4%) hits followed by *Cryptotermes secundus* with 1841 (5.6%) hits (Fig. 1b). Prawn species including *Litopenaeus vannamei, M. nipponense, M. rosenbergii, Penaeus monodon,* and *M. japonicus* were the eighth, eleventh, thirteenth, seventeenth, and eighteenth top-hits, respectively, as demonstrated in top-hit crustaceans species distribution (Fig. 1c). Annotation results of assembled transcriptome are available in Additional file 1: Tables S1 and S2.

### Gene ontology mapping

**Fig. 2:**
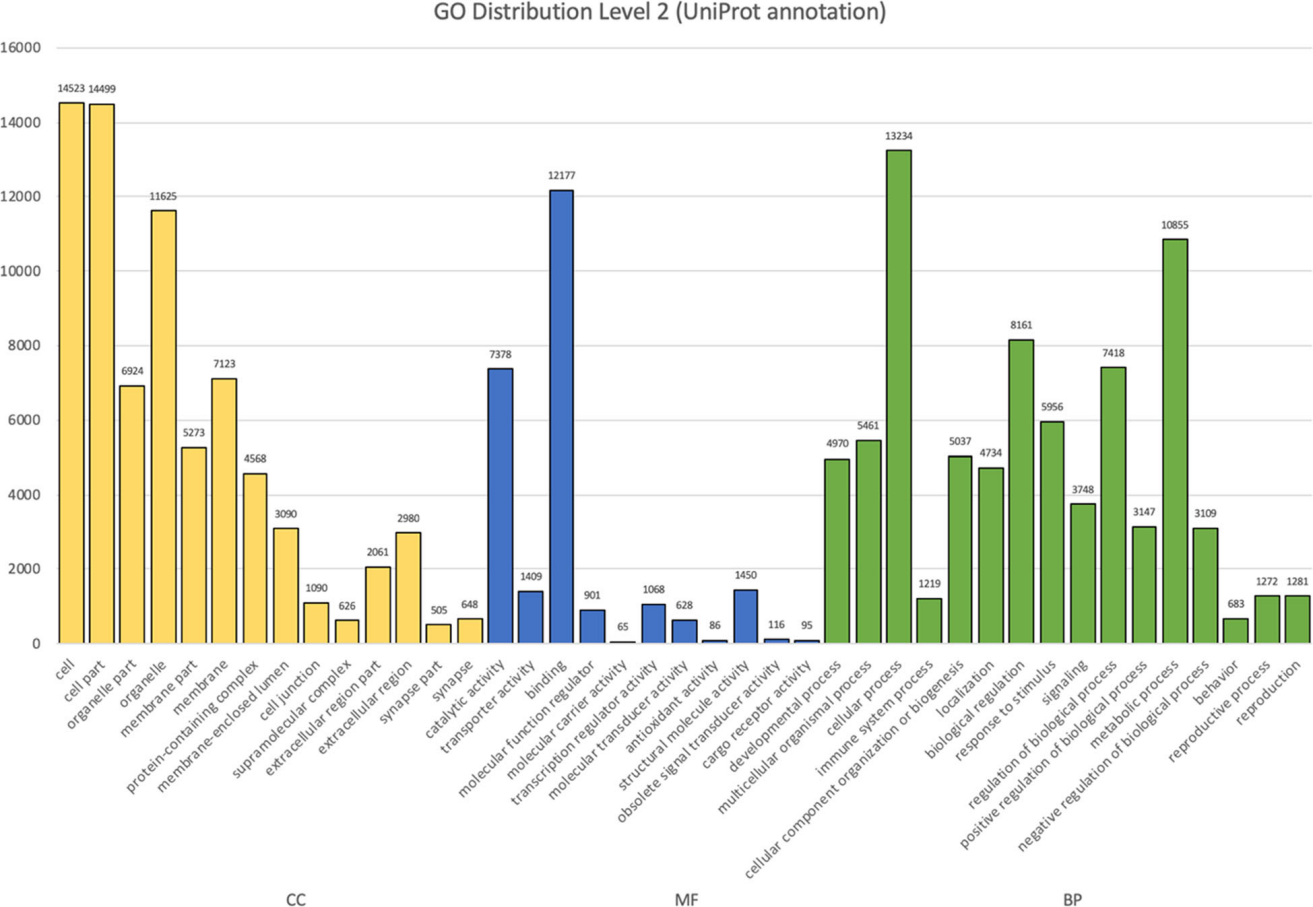
GO distribution (level 2) of annotated unigenes based on UniProt database. GO assignments were divided into three categories including cellular process (CC, yellow), molecular function (MF, blue), and biological process (BP, green)

Functional annotations including EggNOG (Evolutionary Genealogy of Genes: Non-supervised Orthologous Groups), KEGG (Kyoto Encyclopedia of Genes and Genomes), and GO (Gene Ontology) were obtained from Blastx UniProt results using Trinotate suite. Total of 18,291 unigenes were GO mapped. Total of 187,582 GO assignments (level 2) were generated from GO annotations and divided into three GO domains including cellular components (75,535 or 40.27%), molecular functions (25,373 or 13.51%), and biological processes (86,674 or 46.20%) (Fig. 2). Among the cellular component domains, annotated unigenes were mostly involved in “cell” (14,523) and “cell part” (14,499) followed by “organelle” (11,625). The least abundance in cellular component were “synapse part” (505) followed by “supramolecular complex” (626) (Fig. 2). Molecular function domains were primarily dominated by “binding” (12,177) and “catalytic activity” (7378). The least abundance in molecular function were “molecular carrier activity” [35] followed by “antioxidant activity” [36] (Fig. 2). In the biological process domains, annotated unigenes were mostly involved in “cellular process” (13,234) followed by “metabolic process” (10,855). The least abundance in biological process were “behavior” (683) followed by “immune system process” (1219) (Fig. 2).

**Fig. 3:**
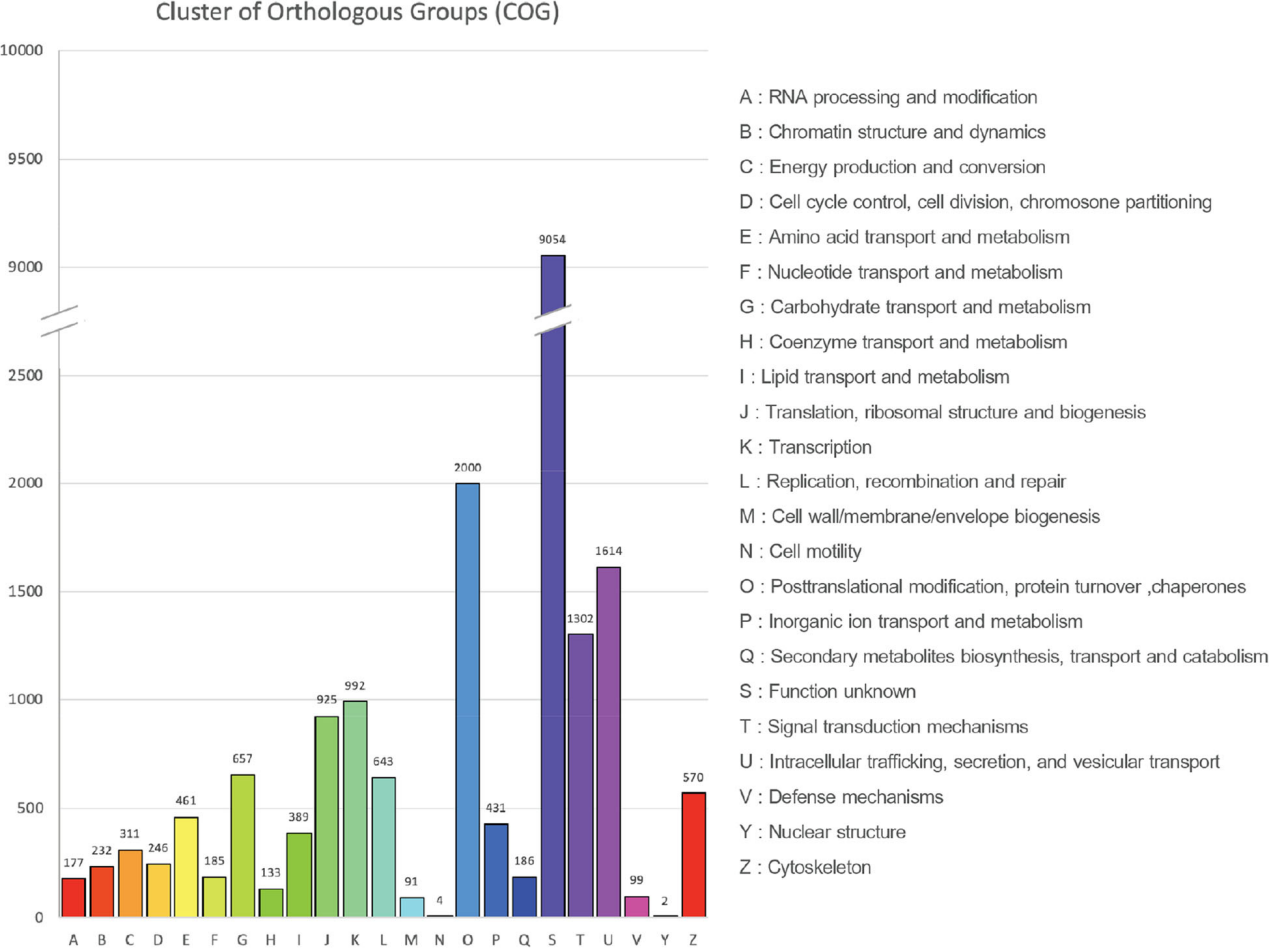
EggNOG classifications of annotated unigenes based on UniProt database. EggNOG functional annotations were divided into 23 categories. The EggNOG categories are shown on the horizontal axis as alphabets with category names on the right

EggNOG classification showed that 20,130 unigenes were identified in the EggNOG database. Total of 20,704 EggNOG functional annotations were obtained and classified into 23 categories (Fig. 3). The most abundant category was “Function unknown” (9054 or 43.73%) The second most abundant was “Post-translational modification, protein turnover, chaperones” (2000 or 9.66%) followed by “Intracellular trafficking, secretion, and vesicular transport” (1614 or 7.79%), and “Signal transduction mechanisms” (1302 or 6.29%), respectively. The least abundant category was “Nuclear structure” (2 or 0.01%) followed by “Cell motility” (4 or 0.02%) (Fig. 3).

**Fig. 4:**
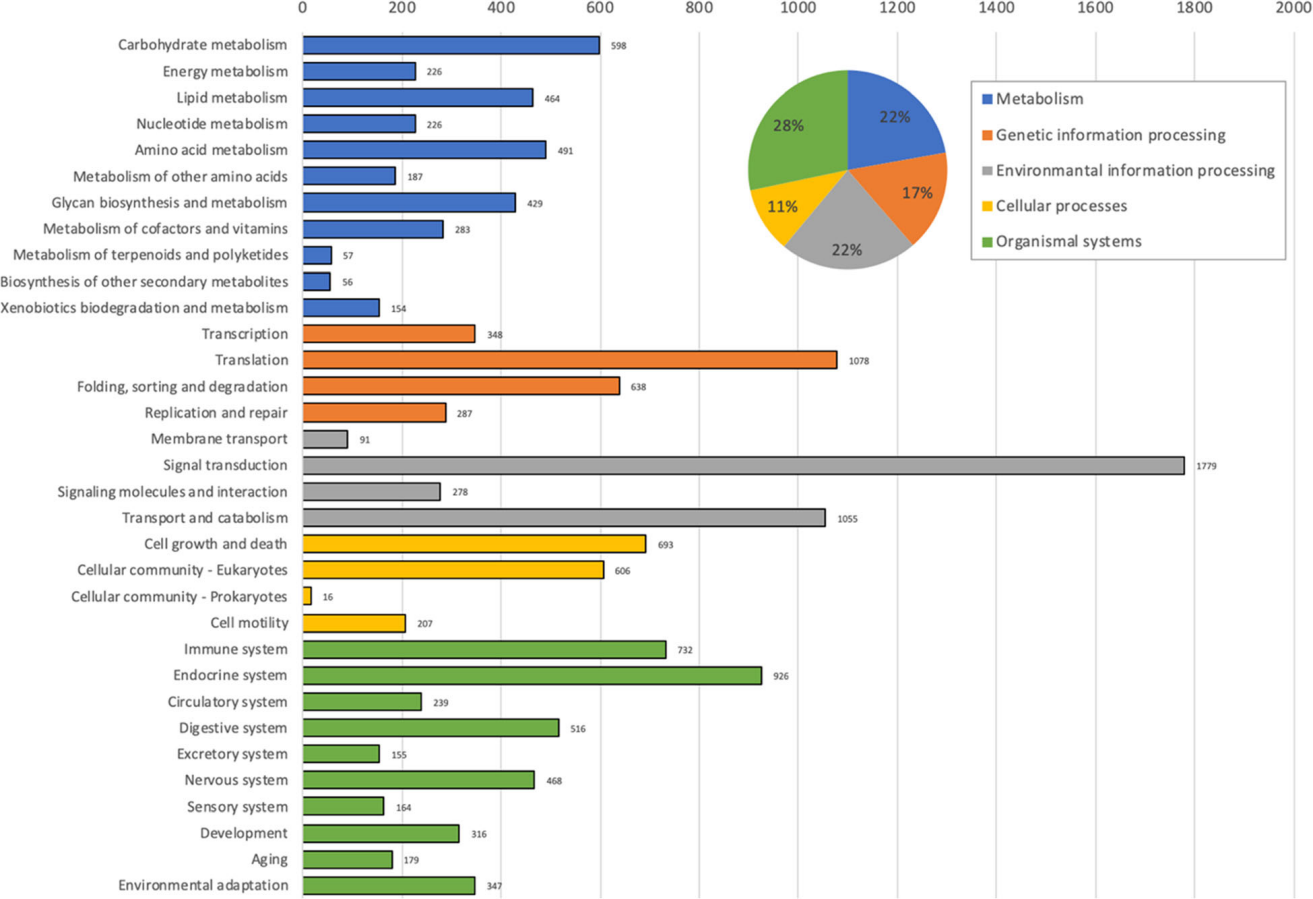
KEGG orthology distribution of annotated unigenes based on UniProt database. KEGG orthology were categorized into five major categories. The names and distribution ratios of each category are shown in pie chart at the top right corner

A total of 19,715 unigenes were matched to the KEGG database. Of those, 7917 unigenes had orthologs in the KEGG orthology database. A total of 14,289 KEGG orthology (KO) were obtained from those unigenes and then categorized into five major categories including “Metabolism” (3171 or 22.19%), “Genetic information processing” (2351 or 16.45%), “Environmental information processing” (3203 or 22.42%), “Cellular processes” (1522 or 10.65%), and “Organismal systems” (4042 or 28.29%) (Fig. 4). KO distribution results showed that the most abundance orthology was signal transduction from “Environmental information processing” category with 1779 unigenes. The second most abundance was translation from “Genetic information processing” category (1078 unigenes) followed by transport and catabolism from “Environmental information processing” category (1055 unigenes), respectively (Fig. 4).

### Differential abundance analysis

To identify differentially abundant transcripts between two groups, transcripts that had count per millions (CPM) more than 1 in at least two samples were selected before the analysis. Total of 31,377 transcripts survived the cut-off and were subjected to TMM normalization. The differential abundance analysis using EdgeR was performed followed by Benjamini-Hochberg method for multiple *p*-value correction. Total of 5538 transcripts were reported to be differentially expressed (FDR < 0.05, LogFC < ±1). Of those, 2413 transcripts were significantly up-regulated and 3125 transcripts were significantly down-regulated after the infection of *Mr*NV. Full-list of differentially abundant transcripts is available in Additional file 2: Table S3. Summary of the transcriptome assembly, annotation and differential abundance analysis were listed in Table 3.

**Table 3:** Summary of the transcriptome assembly, annotation and differential abundance analysis

Total number of contigs	109,616
Total number of unigenes	96,362
NCBI Nr annotated	32,523 (33.75%)
UniProt annotated	25,761 (26.73%)
GO annotated	18,291 (18.98%)
EggNOG annotated	20,130 (20.88%)
KEGG annotated	19,715 (20.46%)
Differentially abundant gene (total)	5538 (FDR < 0.05, LogFC < ±1)
Up-regulated gene	2413
Down-regulated gene	3125

Among those differentially abundant transcripts, various transcripts were reported to be involved in immune system in response to viral infection (Table 4). These transcripts were categorized into 13 functional groups including antiviral protein (1 unigene), antimicrobial protein (9 unigenes), pattern recognition proteins (19 unigenes), toll-signaling pathway (3 unigenes), RNAi pathway (2 unigenes), prophenol oxidase system (4 unigenes), serine proteinase cascade (5 unigenes), ubiquitin proteasome pathway (4 unigenes), antioxidant system (5 unigenes), blood coagulation (2 unigenes), apoptosis (3 unigenes), phagocytosis (7 unigenes), and other immune genes (10 unigenes) as listed in Table 4. From the list, total of 56 unigenes were reported to be significantly up-regulated, whereas 18 unigenes were significantly down-regulated.

**Table 4:** List of DEG transcripts involved in immune system

Unigene	Functional annotation	Organisms	FC
Antiviral protein			
DN14192_c1_g1_i2	antiviral protein	*Litopenaeus vannamei*	2.48
Antimicrobial protein			
DN46855_c0_g1_i1	anti-lipopolysaccharide factor	*Macrobrachium rosenbergii*	2.25
DN3291_c0_g1_i1	anti-lipopolysaccharide factor 1	*Macrobrachium rosenbergii*	2.73
DN34234_c0_g1_i1	anti-lipopolysaccharide factor 3	*Macrobrachium rosenbergii*	4.20
DN9599_c0_g1_i3	crustin 7, partial	*Macrobrachium rosenbergii*	6.59
DN584_c0_g1_i2	crustin 6, partial	*Macrobrachium rosenbergii*	3.48
DN2919_c0_g1_i1	crustin 5	*Macrobrachium rosenbergii*	3.05
DN13113_c0_g1_i1	i-type lysozyme-like protein 2	*Penaeus monodon*	−2.45
DN5315_c0_g1_i1	crustin A	*Litopenaeus vannamei*	−8.94
DN25544_c0_g1_i1	crustin 4	*Panulirus japonicus*	−2.25
Pattern recognition proteins (PRPs)			
DN27838_c0_g1_i1	C-type lectin	*Procambarus clarkii*	3.29
DN3149_c0_g1_i3	C-type lectin 1	*Palaemon modestus*	3.46
DN25495_c0_g1_i4	C-type lectin 2	*Marsupenaeus japonicus*	4.32
DN39427_c0_g1_i1	C-type lectin 4	*Fenneropenaeus merguiensis*	−2.91
DN8487_c0_g1_i1	C-type lectin H	*Eriocheir sinensis*	−3.05
DN79_c0_g1_i9	C-type lectin-like domain-containing protein PtLP	*Portunus trituberculatus*	2.58
DN9664_c0_g1_i1	C-type lectin-like protein	*Fenneropenaeus chinensis*	−4.08
DN1597_c0_g1_i2	down syndrome cell adhesion molecule	*Cherax quadricarinatus*	−2.03
DN1969_c0_g1_i12	ficolin	*Macrobrachium nipponense*	2.55
DN458_c0_g1_i6	ficolin-like protein 2	*Pacifastacus leniusculus*	3.32
DN2732_c0_g1_i1	lectin	*Macrobrachium rosenbergii*	2.41
DN22168_c0_g1_i1	lectin 1	*Macrobrachium rosenbergii*	2.36
DN1016_c0_g1_i2	lectin 2	*Macrobrachium rosenbergii*	3.56
DN2249_c0_g1_i2	lectin 3	*Macrobrachium rosenbergii*	2.28
DN248_c1_g1_i2	lectin B isoform 2, partial	*Marsupenaeus japonicus*	2.13
DN59056_c0_g1_i1	lectin D, partial	*Marsupenaeus japonicus*	2.45
DN11184_c0_g1_i1	lectin E	*Marsupenaeus japonicus*	2.68
DN15806_c0_g1_i1	mannose-binding protein	*Procambarus clarkii*	−6.02
DN51_c0_g1_i3	tachylectin	*Macrobrachium rosenbergii*	2.17
Toll-IMD signaling pathway			
DN1501_c0_g4_i1	spatzle protein, partial	*Fenneropenaeus chinensis*	−17.88
DN6896_c0_g2_i1	toll-receptor 9	*Penaeus monodon*	3.81
DN48150_c0_g1_i1	Nuclear factor NF-kappa-B p110 subunit	*Nicrophorus vespilloides*	4.69
RNAi pathway			
DN14942_c0_g1_i2	dicer-2	*Macrobrachium rosenbergii*	3.66
DN13890_c0_g1_i2	argonaute-3	*Macrobrachium rosenbergii*	2.16
Prophenol oxidase system (ProPO)			
DN24054_c0_g1_i2	prophenoloxidase, partial	*Macrobrachium rosenbergii*	2.01
DN9200_c0_g1_i1	prophenoloxidase-activating enzyme 2a	*Penaeus monodon*	2.99
DN27498_c0_g1_i2	prophenoloxidase activating factor 1	*Scylla paramamosain*	2.31
DN6266_c0_g1_i1	prophenoloxide activating enzyme III	*Macrobrachium rosenbergii*	2.23
Serine proteinase cascade			
DN449_c1_g1_i1	serine proteinase	*Scylla paramamosain*	2.51
DN11492_c0_g1_i8	serine proteinase stubble	*Lucilia cuprina*	−6.73
DN833_c0_g1_i2	serine proteinase inhibitor 6	*Penaeus monodon*	−2.48
DN2466_c0_g1_i2	alpha-2-macroglobulin	*Macrobrachium rosenbergii*	−2.68
DN17466_c0_g1_i2	pacifastin heavy chain	*Macrobrachium rosenbergii*	2.14
Ubiquitin proteasome pathway			
DN49814_c0_g5_i1	E3 ubiquitin-protein ligase Ubr3	*Copidosoma floridanum*	2.22
DN8249_c1_g1_i1	E3 ubiquitin-protein ligase RNF216-like, partial	*Hyalella azteca*	3.89
DN10760_c0_g1_i3	ubiquitin	*Papilio xuthus*	−4.89
DN1685_c0_g1_i9	RING finger protein nhl-1-like	*Limulus polyphemus*	3.63
Antioxidant system			
DN6006_c0_g2_i1	Microsomal glutathione S-transferase 1	*Penaeus monodon*	2.01
DN19734_c0_g1_i1	glutathione peroxidase 3	*Penaeus monodon*	4.08
DN8125_c0_g1_i2	selenium independent glutathione peroxidase	*Penaeus monodon*	−2.20
DN27870_c0_g2_i3	copper/zinc superoxide dismutase isoform 3	*Marsupenaeus japonicus*	−13.45
DN29677_c0_g1_i1	thioredoxin	*Macrobrachium nipponense*	−2.16
Blood coagulation			
DN49188_c0_g1_i1	transglutaminase	*Macrobrachium rosenbergii*	2.08
DN2823_c0_g1_i2	hemicentin-1-like isoform X2	*Pieris rapae*	5.31
Apoptosis			
DN370_c0_g1_i2	caspase	*Eriocheir sinensis*	2.64
DN12951_c0_g1_i2	caspase 4	*Portunus trituberculatus*	3.76
DN21090_c0_g1_i2	inhibitor of apoptosis protein	*Scylla paramamosain*	2.79
Phagocytosis			
DN57596_c0_g2_i1	Ras-related protein Rab-37	*Zootermopsis nevadensis*	2.66
DN13418_c0_g1_i1	Rab32	*Macrobrachium rosenbergii*	2.46
DN19755_c0_g3_i1	rac GTPase-activating protein 1-like	*Centruroides sculpturatus*	2.95
DN13060_c0_g1_i1	VLIG2	*Macrobrachium rosenbergii*	3.92
DN147_c0_g2_i3	VLIG1	*Macrobrachium rosenbergii*	2.89
DN18093_c0_g1_i3	ADP-ribosylation factor	*Marsupenaeus japonicus*	2.19
DN27087_c0_g1_i2	interferon regulatory factor	*Litopenaeus vannamei*	3.32
Other immune genes			
DN8990_c0_g1_i3	Ferritin	*Macrobrachium rosenbergii*	2.93
DN39999_c0_g1_i6	calcium/calmodulin-dependent protein kinase type II alpha chain isoform X6	*Zootermopsis nevadensis*	2.19
DN47919_c0_g1_i9	integrin, partial	*Procambarus clarkii*	2.53
DN17544_c0_g2_i1	integrin alpha 8	*Fenneropenaeus chinensis*	2.55
DN11203_c0_g4_i1	integrin alpha 4, partial	*Fenneropenaeus chinensis*	2.04
DN2727_c0_g1_i3	Cathepsin B	*Macrobrachium rosenbergii*	2.69
DN928_c0_g1_i2	cathepsin C	*Fenneropenaeus chinensis*	2.50
DN12284_c0_g1_i1	cathepsin L	*Marsupenaeus japonicus*	2.66
DN1236_c0_g1_i6	crustacyanin-like lipocalin	*Macrobrachium rosenbergii*	−2.10
DN18816_c0_g2_i1	crustacyanin A, partial	*Penaeus monodon*	−2.48

**Fig. 5:**
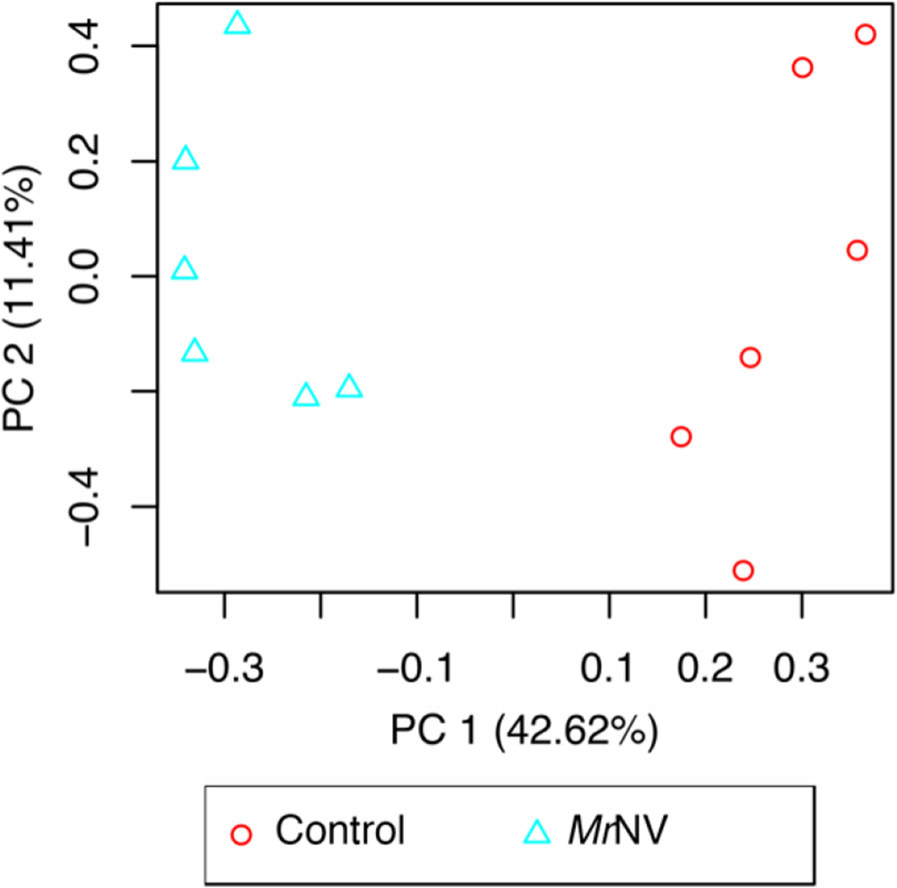
Principle component analysis of twelve samples (84,092 transcripts). PC 1 and 2 are principle component 1 and 2, respectively. Blue triangles are *Mr*NV-infected group (*n* = 6), whereas red circles are control group (n = 6)

**Fig. 6:**
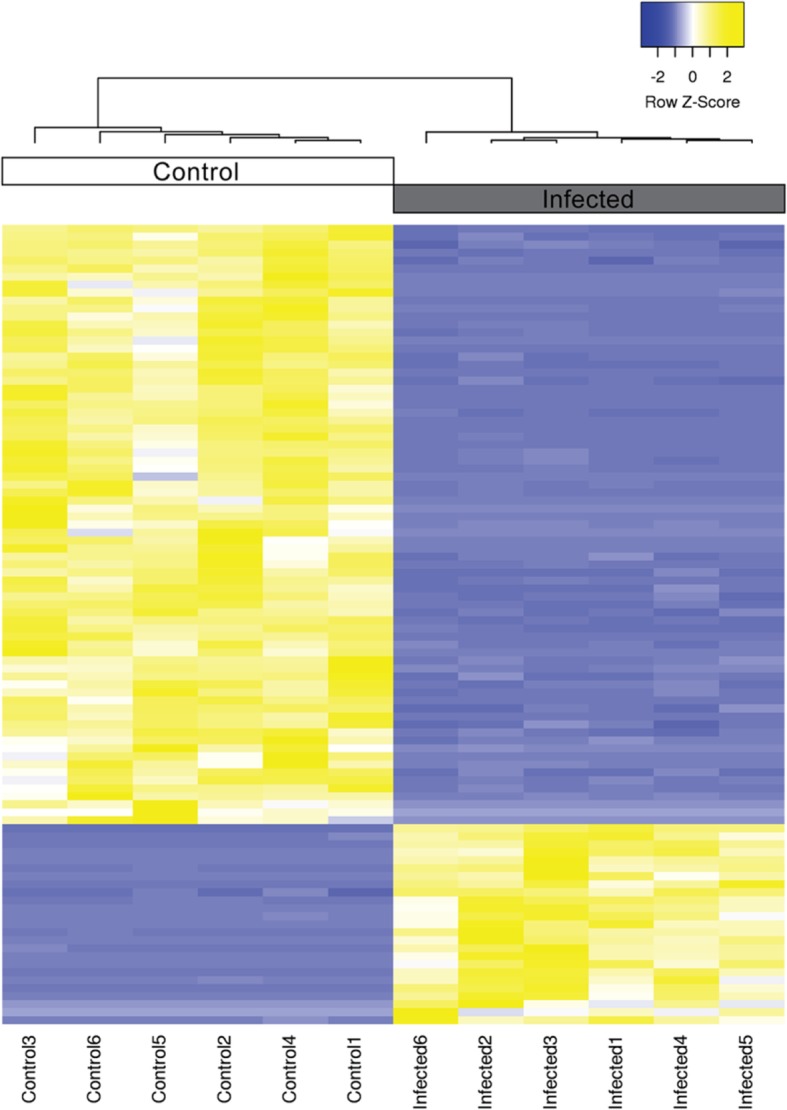
Heatmap of top 100 differentially expressed transcripts. The heatmap was generated using trimmed mean of M-values (TMM). Sample clustering was done using Pearson’s correlation. The Z-score scale is shown in the top-right corner ranging from − 2 (blue) to 2 (yellow)

To examine the homogeneity across biological replicates, principle component analysis (PCA) was performed. Transcripts with extremely low abundance (sum of read count < 10) were filtered out. Total of 84,092 transcripts survived the cut-off and were subjected to the PCA. The PCA results showed strong clustering within each group. The both groups formed distinct clusters within principle component 1 (PC1) which responsible for 42.62% of the variance in the expression (Fig. 5). In addition, the top 100 most differentially abundant transcripts were clustered using Pearson’s correlation and displayed in heatmap (Fig. 6). The biological replicates were clustered within the same group and demonstrate clear distinction between control and infected group.

### Quantitative RT-PCR

**Fig. 7:**
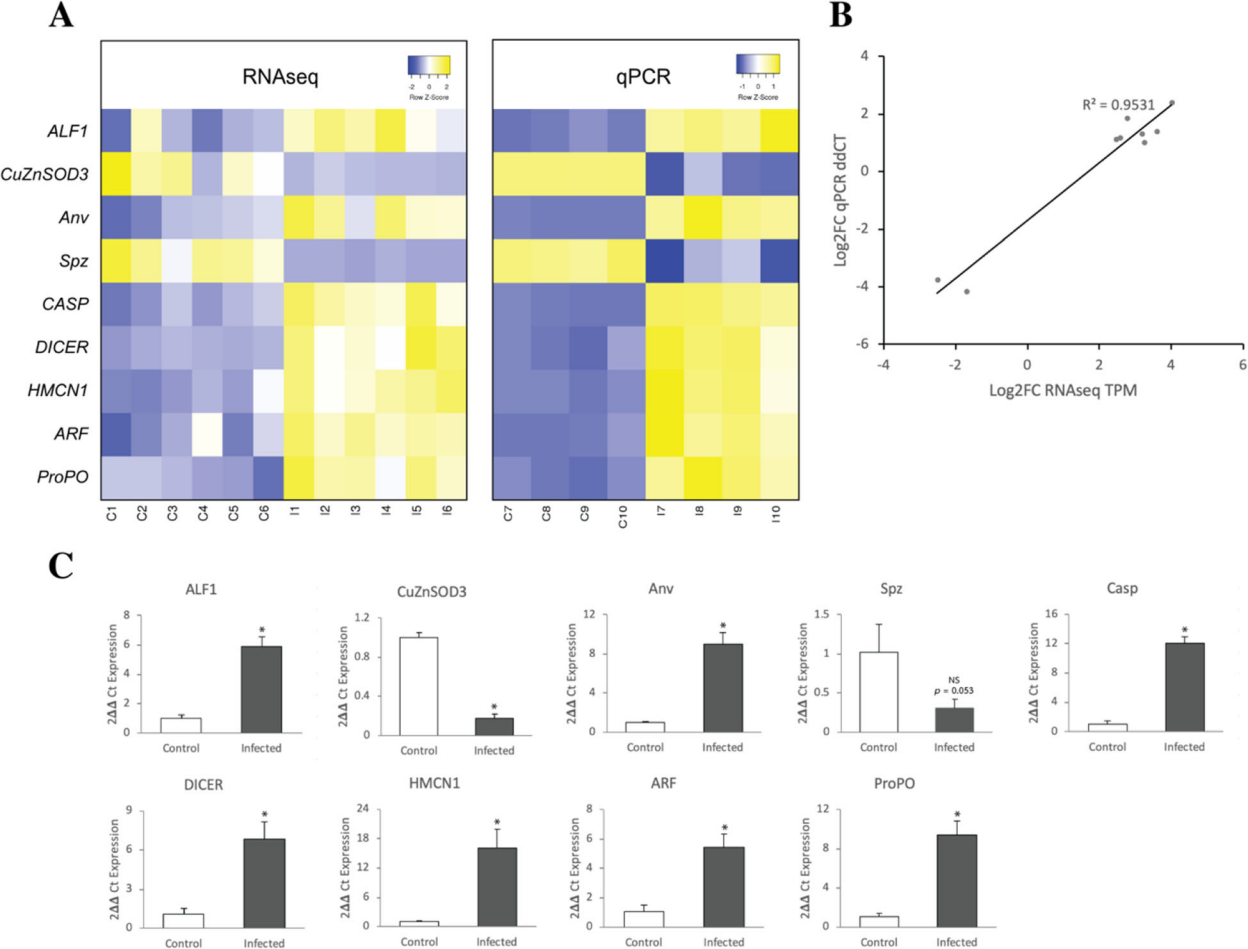
Comparison of fold change in gene expression using either qRT-PCR or RNAseq. **a** Heatmap representing transcript per million (TPM) expression from RNAseq and relative expression from qRT-PCR. The Z-score scales are shown in the top-right corner ranging from blue to yellow. **b** Regression plot demonstrating the direct correlation between the average log2 FC expression values from both RNAseq and qRT-PCR. **c** The qRT-PCR validation results of nine selected genes including *anti-lipopolysaccharide factor 1 (ALF1), Spatzle (Spz), copper/zinc superoxide dismutase 3 (CuZnSOD3), caspase (CASP), antiviral protein (Anv), dicer (DICER), hemicentin-1-like (HMCN1), ADP ribosylation factors (ARF), and prophenoloxidase (ProPO)* with *elongation factor1-alpha (EF1-alpha)* as an internal reference

To validate differential abundance results from the RNAseq pipeline, qRT-PCR was performed using nine selected genes from the list of DEG involved in the immune system. *Elongation factor1-alpha* (EF1-alpha) was used as an internal reference gene. Four separate biological replicates from each group were used in two-step qRT-PCR. The abundance levels were calculated using the delta-delta C_t_ method. The qRT-PCR results showed that all up-regulated genes had greater differences in transcript abundance than those of RNAseq, whereas all down-regulated genes had smaller differences (Table 5). According to the qRT-PCR results, all of the selected genes were differentially abundant after the infection of *Mr*NV (*p* > 0.05) except *Spz* which had *p*-value of 0.053 (Table 5). Comparative heatmaps were generated using relative abundance (qRT-PCR) and transcript per millions (TPM) for RNAseq results (Fig. 7a). Expression patterns of all selected genes were comparable between qRT-PCR and RNAseq. Furthermore, the Pearson’s correlation coefficients were calculated using the average log-fold change ratio between the two methods and demonstrated highly significant correlation as shown in Fig. 7b (R^2^ = 0.9531).

**Table 5:** Comparison of fold change in transcript abundance between qRT-PCR and RNAseq

Gene symbol	qRT-PCR		RNAseq	
	Fold change ± SD	*P*	Fold change	Corrected P
*ALF1*	5.86 ± 0.72	0.001	2.25	0.011
*CuZnSOD3*	−5.72 ± 1.09	0.003	−13.45	0.001
*Anv*	8.97 ± 1.19	0.001	2.48	0.007
*Spz*	−3.26 ± 1.08	0.053	−17.88	2.90E-09
*CASP*	12.05 ± 0.91	7.04E-05	2.64	1.03E-06
*DICER*	6.81 ± 1.32	0.006	3.66	4.31E-11
*HMCN1*	16.07 ± 3.91	0.005	5.31	0.003
*ARF*	5.45 ± 0.90	0.004	2.19	0.008
*ProPO*	9.38 ± 1.43	0.002	2.01	8.65E-06

## Discussion

In this study, we report a highly complete transcriptome for *M. rosenbergii* post-larvae and identified immune-related genes in response to *Mr*NV infection using NGS platform as well as verified the transcript abundance of selected genes using qRT-PCR. We not only present the transcriptome data, but we have published our computational pipeline to benefit the wider scientific community. Raw data has been uploaded to the National Centre for Biotechnology Information Sequence Read Archive (SRA) under the accession BioProject number: PRJNA550272. The data analysis pipeline is available on GitHub at https://github.com/prawnseq/Mrosenbergii_MrNV_RNAseq.

The transcriptome of giant fresh water prawn (*M. rosenbergii*) was assembled to expand the transcriptomic resources for further gene expression analysis of this species. Therefore, we aimed for maximizing transcriptome coverage while minimizing the redundancy to generate high quality transcriptome. The BUSCO results showed that the assembled transcriptome was highly complete (C:83.4%) with low fragmentation (F:13%), missing (M:3.3%), and duplication (D:16.2%). These results were comparable to the assembled transcriptome from *L. vannamei* (C:98.0%, F:0.7%, M:1.3%, D:25.5%) [37] and from *P. monodon* (C:98.2%, F:0.8%, M:1.0%, D:51.3%) [20]. According to Blastx results against UniProt database, the top hits were mammalian species (*H. sapiens, M. musculus*) followed by *D. melanogaster* because UniProt database is a comprehensive annotation database which annotated and reviewed by the experts [38]. Therefore, majority of UniProt subjects were obtained from well-studied model organisms such as human, mouse and fruit fly. However, according to Blastx results against Nr-Arthropod database, majority of the top hits were matched to crustacean species, *Hyalella Azteca*. Previous transcriptomic studies of *M.rosenbergii* performed de novo assembly separately from each sample and then clustered into a global transcriptome [30–33]. In this study, all read data were merged before the assembly, resulting in an increase in the abundance of low expressed transcripts, therefore increasing coverage of the assembled transcripts [39]. In addition, the highest E_x_N_50_ value at 94% indicated that the assembled transcripts had high coverage and was assembled from sufficient read data [40].

To identify differentially abundance transcripts associated with *Mr*NV infection, we performed RNA sequencing on six replicates of each healthy PL and experimentally *Mr*NV infected PL. The results showed that 5538 transcripts were differentially expressed with 2413 transcripts were up-regulated and 3125 transcripts were down-regulated. Among those transcripts, some of these were involved in the innate immune system in response to viral infection.

To validate our transcript assembly and differential abundance results, we also selected 9 targets for validation with qPCR, and demonstrated a very low false discovery rate in these genes. We filtered our list of DEGs according to functional groups of importance including pattern recognition proteins (PRPs) and antiviral protein, prophenol oxidase (ProPO) system, the Toll-IMD signaling pathways, antimicrobial peptides (AMPs) and blood clotting system, phagocytosis and apoptosis, antioxidant system, and RNA interference (RNAi). Numerous studies demonstrated high correlation between RNAseq and qPCR data [41–43]. Moreover, RNAseq studies of *M. rosenbergii* in response to bacterial and viral infection also showed high correlation between RNAseq and qPCR results [30–32]. To enhance our confidence, we validated these results in independent biological samples.

Crustacean innate immune system requires the recognition of the pathogens by pattern recognition proteins (PRPs) to trigger the immune responses [44]. PRPs are groups of germ-line encoded proteins that can activate humoral and cellular immune response via immune signaling pathway [45]. Several studies identified prawn PRPs and examined its role in prawn immune system regarding viral infection. *M. rosenbergii* C-type lectin (*Mr*CTL) expression in hepatopancreas was up-regulated after a challenge with *Vibrio parahaemolyticus* or white spot syndrome virus (WSSV) [46]. Ficolin expression in *M. rosenbergii* hepatopancreas was found to be up-regulated after the infection of *V. anguillarum* and WSSV [47]. Moreover, *M. rosenbergii* mannose-binding lectin (MBL) expression in gills was also up-regulated in response to WSSV or *Mr*NV infection [48]. In this study, various PRPs such as C-type lectin, ficolin, and antiviral protein which are part of C-type lectin family were differentially expressed after the infection of *Mr*NV suggesting that these PRPs play important roles in immune system against *Mr*NV infection. Importantly, we validated the expression of one of these genes, antiviral protein, which was up-regulated by 2.48-fold in the RNAseq and 8.97-fold by qPCR (Table 5). However, in our results, MBL expression was down-regulated which contradicts the previous report [48]. This maybe because MBL is not required for antiviral response of post-larvae prawn against *Mr*NV or the expression of MBL in post-larvae prawn being suppressed by *Mr*NV. Further investigation is needed to understand the effects of *Mr*NV to MBL expression in post-larvae stage.

Prophenol oxidase (ProPO) activating system participates in first line of immune system by triggering melanization and other responses such as hemocyte induction, encapsulation, and nodule formation [14, 49, 50]. Recognition of PAMPs by PRPs leads to activation of serine proteinases cascade, resulting in the production of active PO enzyme. The active PO enzyme produces polymeric melanin around invading pathogens resulting in melanization [50]. The RNAseq presented here shows a 2.01-fold up-regulation of ProPO expression in response to *Mr*NV infection in which the expression was validated by qPCR (9.38-fold up-regulation; Table 5). In addition, three types of prophenoloxidase activating enzyme were also up-regulated in RNAseq results. These results indicate involvement of ProPO-activating system in *Mr*NV infection.

The Toll-IMD signaling pathways are considered to be the most crucial immune signaling pathways in invertebrates [51]. The toll receptor is triggered by cytokine-like ligand Spätzle whereas vertebrate Toll-like receptors (TLRs) recognize pathogens directly [52, 53]. Triggering the toll receptor leads to activation of NF-kappa-B family protein Dif/Dorsal and then leads to up-regulation of immune-related genes such as antimicrobial peptide (AMPs) [53]. Toll receptor from *M. rosenbergii* have been identified [54, 55] and was found to be gradually up-regulated in gills during the WSSV challenge [55]. *M. rosenbergii* spätzle protein has been found to be up-regulated in hemocytes after the bacteria infection [56]. In addition, spätzle protein in *F. chinensis* was up-regulated after challenged with *V. anguillarum* and WSSV [57]. Nuclear factor NF-kappa-B p110,also known as relish, is an important nuclear transcription factor in the IMD signaling pathway which functions parallel to the Toll pathway [53, 58]. *M. rosenbergii* relish was reported to be involved in bacterial infection and overexpression of relish induced the expression of various AMPs [59]. In this study, we found that the toll and NF-kappa-B p110 expression were up-regulated after the infection of *Mr*NV, whereas the expression of spätzle was down-regulated. We also validated the expression of spätzle using qPCR which was 3.26-fold down-regulated compared to 17.88-fold down-regulated in RNAseq (Table 5). These results suggested that these genes are involved in the immune system against *Mr*NV infection. Down-regulation of spätzle may be caused by a negative feedback regulation from the activation of the toll pathway [60, 61].

Antimicrobial peptides (AMPs) are also important components in first line of immune system. AMPs are usually small cationic, amphipathic, germ-line encoded proteins that have rapid and efficient antimicrobial effects against broad spectrum of microorganisms including bacteria, fungi, and viruses. AMPs differ in structural conformation, charge, and amphipathicity [62, 63]. Fundamentally, AMPs disrupt the membrane integrity of the target and then destroy the microbe by membrane destabilization or pore formation [62, 64]. Several studies reported that anti-lipopolysaccharide factors (ALFs) and lysozymes expression were up-regulated after the infection of WSSV in *M. rosenbergii, F. chinensis*, and *L. vannamei* [35, 65, 66]. Crustin expression in *M. rosenbergii* hemocytes was found to be up-regulated after WSSV, infectious hypodermal and hematopoietic necrosis virus (IHHNV), and *Aeromonas hydrophila* challenges [67]. However, crustin isoform 1 and 2 expression in *P. monodon* were down-regulated after WSSV infection, whereas crustin isoform 3 was up-regulated [68]. In our report, ALFs, i-type lysozyme-like protein 2 (LYZL2), and crustin members were differentially expressed during *Mr*NV infection. ALFs and LYZL2 were up-regulated, whereas several crustin isoforms were both up- and down-regulated. Additionally, the validation by qPCR showed that ALF1 was up-regulated by 5.86-fold which was comparable to the 2.25-fold up-regulation by RNAseq (Table 5). These suggested that ALFs and LYZL2 are involved in the prawn immune response against *Mr*NV, whereas only certain isoforms of crustin are involved.

Blood clotting is a humoral response which prevents hemolymph loss and microbial spread during injury [69]. In crustacean, blood clotting involves cross-linking aggregation of clotting proteins (CPs) by calcium-dependent transglutaminase (TGase) produced by the hemocyte [70]. Previous studies showed that lysozyme and crustin expression in *M. japonicus* were down-regulated in TGase depleted prawn suggesting that there is a link between the blood clotting system and AMPs [71]. We found that transglutaminase and hemicentin-1-like isoform X2 (HMCN1) expression were up-regulated. In addition, we validated the expression of HMCN1 which was up-regulated by 5.31-fold in the RNAseq and 16.07-fold by qPCR (Table 5). These results suggested that these two blood coagulation components are involved as a response to the infection of *Mr*NV. Moreover, up-regulation of two blood coagulation components, lysozyme, and certain isoforms of crustin may indicate a link between the blood clotting system and AMPs in *M. rosenbergii*.

Apoptosis or programmed cell death plays an important role in cellular immune response by limiting viral replication and eliminating virus-infected cells in multicellular organisms [16, 72]. Apoptosis requires the activation of caspases, highly conserved cysteine proteases that are involved in the execution of cell death [73]. Previous studies characterized *M. rosenbergii* caspase (*Mr*Casp) and demonstrated that the capsid protein of *Mr*NV could inhibit apoptotic responses of *Mr*Casp in Sf9 cells [74]. *M. rosenbergii* caspase 3c expression in hemocyte was found to be up-regulated after IHHNV challenge [75]. *M. rosenbergii* inhibitor of apoptosis protein (IAP) has been characterized and was found to be up-regulated in hepatopancreas after IHHNV infection [76]. IAP is one of the apoptosis regulators that binds and inhibits the activity of caspase [77]. Additionally, *L. vannamei* IAPs-silenced by RNAi demonstrated higher expression of WSSV genes and significant down-regulation of AMP genes [35]. In this study, we reported up-regulation of caspase, caspase4, and IAP in response to *Mr*NV challenge. We also validated the expression of caspase which was 2.64-fold up-regulation in RNAseq and 12.05-fold by qPCR (Table 5). Up-regulation of these genes suggests that these gene are involved in *Mr*NV infection.

Phagocytosis plays a role in innate immune system by ingesting microparticles including microbial pathogens and cellular debris from apoptosis and necrosis [78]. Recent studies reported that the small GTPases play a role in antiviral immunity by controlling cellular trafficking and regulating phagocytosis [79–81]. ADP ribosylation factors (Arfs), which are small ubiquitously expressed GTPases have been characterized in *M. rosenbergii* and *M. japonicus.* Arfs expression was up-regulated in both species after WSSV challenge [82, 83] [84]. The expression of Rab GTPases, Ras-like GTPase superfamily members, are up-regulated in response to WSSV [85] and *MrNV* infection. We validated, ADP ribosylation factors (Arfs), using qPCR and found 5.45-fold up-regulation compared to 2.19-fold by RNAseq (Table 5). These indicates involvement of these small GTPases in antiviral immunity against *Mr*NV.

Antioxidant enzymes are responsible for removing harmful reactive oxygen species (ROS) during the prawn immune response [86]. Several antioxidant enzymes have been identified in *M. rosenbergii* such as selenium dependent glutathione peroxidase [36], glutathione S-transferase (GST) [87], copper/zinc superoxide dismutase (CuZnSOD) [88], and thiol-dependent peroxiredoxin (Prdx) [89]. These genes have been reported to be differentially expressed according to different types of pathogen [36, 87–89]. We identified five antioxidant enzyme genes that were differentially expressed during *Mr*NV infection. We also validated the expression of one of these genes, CuZnSOD3, using qPCR which was 5.72-fold down-regulated compared to 13.45-fold down-regulated in RNAseq (Table 5).

In prawns, RNA interference (RNAi) plays a crucial role in antiviral immunity. Several key components of RNAi has been characterized in prawns including isoforms of dicer and argonaute in *M. rosenbergii* [90], *L. vannamei* [91–93], and *P. monodon* [94, 95]. Dicer, RNase III-like enzyme, is responsible for the cleavage of long dsRNA into 21–30 bp siRNA which initiates RNAi mechanism [96, 97]. Genome derived silencing RNAs (siRNAs) are unwound and incorporated with argonaute protein which is the main component of the RNA-induced silencing complex (RISC) [98]. RISC then recognizes and degrades target mRNA which represses the expression of the target genes [99]. Previous studies reported that administration of synthetic dsRNA/ siRNA specific to genes of yellow head virus (YHV) [100, 101], or WSSV [102] resulted in specific inhibition of viral replication. Moreover, dsRNA can be formed during the replication of both RNA (taura syndrome virus (TSV) and YHV) and DNA viruses (WSSV) which triggers antiviral responses via RNAi [103]. Recent studies revealed that 31 miRNAs were differentially expressed during WSSV infection suggesting that these miRNA may be involved in innate immunity [104]. Additionally, a total of 24 miRNAs have been identified in *M. japonicus* and reported that these miRNAs may be involved in innate immunity including regulating processes such as phagocytosis, proPO system, and apoptosis [105]. Taken together, both siRNAs and miRNAs have participated in shrimp antiviral immunity. In this study, dicer-2 and argonaute-3 were significantly up-regulated in response to the *Mr*NV infection. Importantly, the qPCR validation showed 6.81-fold up-regulation of dicer-2 compared to 3.66-fold up-regulation from RNAseq results (Table 5) indicating that the production of RNAi may play an antiviral role against *Mr*NV infection in *M. rosenbergii*.

## Conclusion

In conclusion, this study reported a highly complete transcriptome from the post-larvae stage of giant river prawn, *M. rosenbergii.* This transcriptome expands the transcriptomic resources for further gene functional analysis and transcriptome profiling in response to certain conditions. Transcripts abundance analysis between control and *Mr*NV-infected group revealed significant differences in the transcript abundance of various immune responses e.g. immune signaling pathway, prophenol oxidase system, antimicrobial peptides, blood clotting system, phagocytosis and apoptosis, and RNA interference. To our knowledge, this study is the first report of the transcriptomic profile of *M. rosenbergii* post-larvae during the infection of *Mr*NV. This study also provides preliminary insight on molecular responses of the prawn to *Mr*NV infection that improve our understanding of *M. rosenbergii* antiviral responses and may provide molecular targets to help contain the disease outbreak.

## Materials and methods

### Preparation of *Mr*NV infected *M. rosenbergii* post-larvae

*M. rosenbergii* post-larvae (PL 25–30) were purchased from local farm at Suphan Buri provinces, Thailand. The healthy PL were kept in a glass tank with dechlorinated freshwater and continuous aeration at room temperature (25–27 °C). After 1 day of acclimation, the PL were divided into two groups including control and *Mr*NV group. In the *Mr*NV group, the PL were challenged with 10% w/v *Mr*NV-infected PL homogenated in TN buffer (20 mM Tris-HCl and 0.4 M NaCl, pH 7.4) using the immersion method [106]. The control group were challenged with TN buffer instead of *Mr*NV. After 4 days of immersion, the *Mr*NV-infected PL and the PL from control group were divided into 10 subgroups (10 PL per each subgroup). Six subgroups were used in Illumina sequencing, whereas four subgroups were used in quantitative RT-PCR experiment. The PL samples were immersed in DNA/RNA Shield (Zymo Research, USA) and stored at -80 °C until RNA extraction.

The PL from both groups were subjected to *Mr*NV detection using RT-PCR. Viral nucleic acid was extracted from the PL using High Pure Viral Nucleic Acid Kit (Roche). The *Mr*NV-specific primers designed by Senapin and others were used in this study [107]. The RT-PCR was carried out using SuperScript® III One-Step RT-PCR System (Invitrogen) and the extracted viral nucleic acid as a template. The RT-PCR condition was 50 °C for 30 min, followed by 94 °C for 5 min, 35 cycles of 94 °C for 1 min, 55 °C for 45 s, and 72 °C for 1 min, and final extension at 72 °C for 10 min.

### RNA extraction and Illumina sequencing

Total RNA was extracted from each subgroup using Quick-RNA™ MiniPrep (Zymo Research, USA) according to manufacturer’s protocol. The extracted RNA was quantified using DropSense 16 Micro-volume spectrophotometer (Unchained Labs, USA) and stored at -80 °C until library preparation.

The cDNA library was prepared using SENSE mRNA-Seq Library Prep Kit V2 (Lexogen, USA) and the purification module with magnetic beads (Lexogen, USA). Each library was indexed by 6-nucleotide-long i7 indices during library amplification step according to manufacturer’s protocol. The prepared cDNA library was subjected to quality assessment using Qubit 4 Fluorometer (Invitrogen, USA) and LabChip GX Touch 24 microfluidic nucleic acid analyzer (PerkinElmer, USA). Before denaturation, equal amount of each cDNA library were pooled together and re-purified using the magnetic beads. Total of 2 nM pooled library was subjected to denaturation steps according to NextSeq System Denature and Dilute Libraries Guide (Illumina, USA). Cluster generation and paired-end sequencing with 75 bp were performed on a NextSeq 550 sequencer (Illumina, USA) using NextSeq 500/550 High Output Reagent Cartridge v2 150 cycles (Illumina, USA).

### Data analysis pipeline

**Fig. 8:**
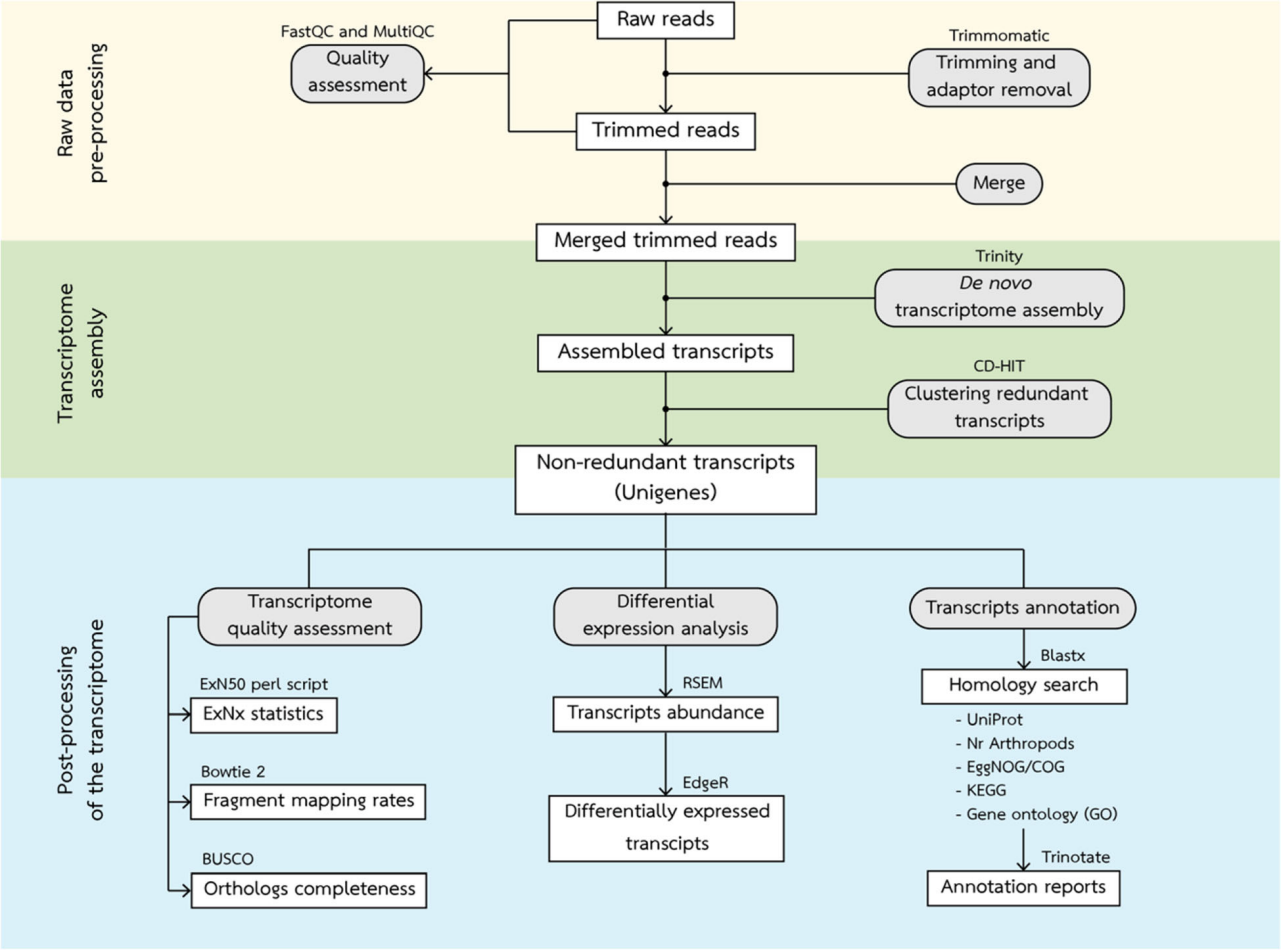
The overviews of analysis pipeline. The pipeline is divided into three major parts including raw data pre-processing, transcriptome assembly, and post-precessing of the transcriptome which indicated by area of different colors. Boxes represent datasets. Rounded boxes represent analyses. The software for each analysis is indicated at the top of the box whereas the database for homology search are listed under the box

The automated data analysis pipeline was written in python using the Snakemake tool [108]. The data analysis pipeline contained three major sections including raw data pre-processing, transcriptome assembly, and post-processing of the transcriptome (Fig. 8). Quality assessment of the sequence data was performed using FastQC v. 0.11.5 [109] and complied using MultiQC v 1.8 [110]. Raw read data were subjected to quality trimming including trim low quality bases, removing N nucleotides, and discarding reads below 36 bases long using Trimmomatic v 0.36 [111]. The trimmed reads were subjected to quality assessment using FastQC and MultiQC and then merged for transcriptome assembly using merge command.

The de novo transcriptome assembly was performed using Trinity software v 2.8.0 using default parameters [112]. The *in-silico* normalization was performed within Trinity prior to de novo assembly. This process resulted in transcripts with relatively high redundancy. To remove the redundancy, transcripts that have more than 95% of identity were clustered together using CD-HIT software [113]. The final transcriptome was subjected to quality assessment including calculate the fragment mapping rates using Bowtie 2 v 2.3.0 [114], examine orthologs completeness using BUSCO v 3 [115] against arthropoda_odb9 database, and generate N_x_ and E_x_N_50_ statistics using ‘contig_ExN50_statistic.pl’ script within Trinity assembler.

The final transcriptome was aligned against UniProt and NCBI’s non-redundant arthropod database using Blastx. The results from Blastx against UNIPROT database were then used to obtain functional annotation from EggNOG, KEGG, and GO database using Trinotate v 3.0.2 [40].

To identify differentially abundant transcripts, the raw read counts were calculated by RSEM software [116] and then used to generate abundance matrix. Differential abundance analysis was performed using EdgeR [117]. EdgeR uses the trimmed mean of M-values normalization method (TMM) to calculate the transcript abundance levels [118] with the Benjamini-Hochberg method for multiple *p*-value correction [119]. The transcripts that had at least two-fold change with a false discovery rate (FDR or adjusted *p*-value) less than 0.05 was considered as differentially abundant transcripts.

### Quantitative RT-PCR analysis

Nine differentially abundant unigenes were selected for quantitaltive RT-PCR (qRT-PCR) including *anti-lipopolysaccharide factor 1 (ALF1), Spatzle (Spz), copper/zinc superoxide dismutase 3 (CuZnSOD3), caspase (CASP), antiviral protein (Anv), dicer (DICER), hemicentin-1-like (HMCN1), ADP ribosylation factors (ARF), and prophenoloxidase (ProPO).* In this study, *elongation factor1-alpha (EF1-alpha)* were used as an internal reference gene [120]. Primers used in the qRT-PCR experiments were listed in Table 6. Primers were subjected to primer efficiency testing using two-fold dilution of cDNA ranging from 200 ng to 12.5 ng. The efficiency of primers was calculated using the following formula “Efficiency (100%) = (10^(-1/ Slope)-1)*100”. Primers with efficiency score between 90 and 110% were used in the qRT-PCR validation.

**Table 6:** Primers used in the qRT-PCR experiment

Primer name	Sequence	Efficiency (%)	R^2^
ALF1-F	5′-CTG GTG ACG GAA GAA GAA GC-3′	98.13	0.9975
ALF1-R	5′–CTT AAC CAG GCC ATT CCT CA–3’		
Spz-F	5′-CGA CGGA ATA CCC GAC CTA CA-3’	92.27	0.9926
Spz-R	5′-TGT CGG TTT TGC AGA CGT AG-3’		
CuZnSOD3-F	5′-GGG AGA CCT AGG GAA CAT CC-3’	95.33	0.9920
CuZnSOD3-R	5′-GTG GAT GAC CAC GGC TCT AT-3’		
CASP-F	5′-CTG CCC TGA ATT CCT CTC TG-3’	105.24	0.9885
CASP-R	5′-CGA AGG TGG TAT GGA GCA AT-3’		
Anv-F	5′-AAT GGT GGT ATC AGC CTT GC-3’	94.53	0.9885
Anv-R	5′-TTA GAG GGT CGA CCA TGA GG-3’		
DICER-F	5′-CAC TCG AGC ATC CTG TTT CA-3’	107.80	0.9969
DICER-R	5′-ACC AAT CCC CAT CCA ATG TA-3’		
HMCN1-F	5′-TAA GGC AAC CGA CCA CTA CC-3’	107.85	0.9940
HMCN1-R	5′-GAC GTA GAG ACT GGC GGA AG-3’		
ARF-F	5′-CCC ATT ACA GTG GTC CTG CT-3’	95.22	0.9928
ARF-R	5′-CAG AAC CCT TCC CTT CAC AA-3’		
ProPO-F	5′-AAC AAC CTG AGA ACC GGA TG-3’	93.46	0.9899
ProPO-R	5′-CGG CAG GGT TGG CAT AAT CT-3’		
EF1a-F	5′-TGC GCT GTG TTG ATT GTA GC-3’	103.19	0.9834
EF1a-R	5′-ACA ATG AGC TGC TTG ACA CC-3’		

Total RNA was extracted from separate biological samples using Quick-RNA™ MiniPrep (Zymo Research, USA). The first-strand cDNA was synthesized using SensiFAST™ cDNA Synthesis Kit (Bioline, UK) according to manufacturer’s protocol. The qPCR was carried out using SensiFAST™ SYBR® Lo-ROX Kit (Bioline, UK). The qPCR condition was 95 °C for 2 min, 40 cycles of 95 °C for 5 s, 60 °C for 10 s, and 72 °C for 15 s, followed by melting curve analysis. The delta-delta C_t_ method was used to calculate relative fold-change of gene expression between control and infected samples [121]. Statistical differences between two groups were conducted using a simple student t-test. The results from qPCR were then compared with the results from RNAseq pipeline using heatmap and coefficient of determination (R^2^).

## Supplementary information

**Additional file 1: Table S1.** Annotation results of assembled transcriptome against UniProt database. **Table S2.** Annotation results of assembled transcriptome against Non-redundant Arthropod database.

**Additional file 2: Table S3.** Full-list of differentially expressed transcripts using EdgeR.

## Data Availability

Raw data has been uploaded to the National Centre for Biotechnology Information Sequence Read Archive (SRA) under the accession BioProject number: PRJNA550272. The data analysis pipeline is available on GitHub at https://github.com/prawnseq/Mrosenbergii_MrNV_RNAseq. Annotation results and full-list of differentially expressed transcripts are available in Additional file 1: Tables S1 and S2 and Additional file 2: Table S3.

## Abbreviations

AMPs: Antimicrobial peptides
cDNA: Complementary deoxyribonucleic acid
CPM: Count per millions
EggNOG: Evolutionary Genealogy of Genes: Non-supervised Orthologous Groups
FDR: False discovery rate
GO: Gene Ontology
IHHNV: Infectious hypodermal and hematopoietic necrosis virus
KEGG: Kyoto Encyclopedia of Genes and Genome pathway
LogFC: Log fold change
miRNA: Micro ribonucleic acid
mRNA: Messenger ribonucleic acid
*Mr*NV: *Macrobrachium rosenbergii* nodavirus
NCBI: National Center for Biotechnology Information
NGS: Next generation sequencing
PAMPs: Pathogen-associated molecular patterns
PC1: Principle component 1
PCA: Principle component analysis
PL: Post larvae
ProPO: Prophenol oxidase
PRRs: Pattern recognition receptors
qRT-PCR: Quantitative reverse transcription polymerase chain reaction
RdRp: RNA-dependent RNA polymerase
RNAi: RNA interference
RNAseq: Ribonucleic acid sequencing
siRNA: Small interfering ribonucleic acid
SRA: Sequence Read Archive
TMM: Trimmed mean of M-values normalization method
TPM: Transcript per millions
WSSV: White spot syndrome virus
WTD: White tail disease
